# Numerical Evaluation and Experimental Test on a New Giant Magnetostrictive Transducer with Low Heat Loss Design

**DOI:** 10.3390/mi12111397

**Published:** 2021-11-14

**Authors:** Zhuan Bai, Zonghe Zhang, Ju Wang, Xiaoqing Sun, Wei Hu

**Affiliations:** 1 Department of Mechanical Engineering, Donghua University, Shanghai 201620, China; dhubz3017@163.com (Z.B.); wj1698103@163.com (J.W.); 2 Department of Public Affairs, Law School, Shanghai University of International Business and Economics, Shanghai 201620, China; zhangzonghe0709@163.com; 3 State Key Laboratory of Mechanical System and Vibration, Shanghai Jiao Tong University, Shanghai 200240, China; sjtuweihu@163.com

**Keywords:** giant magnetostrictive transducer, heat loss, excitation coil, magnetic circuit optimization, experimental study

## Abstract

Giant magnetostrictive transducer with micro and nano precision has a wide application prospect in the field of remote sensing. However, excessive heat loss of components could generate during the energy conversion and transfer from electric energy to magnetic energy, and magnetic energy to mechanical energy, thereby affecting its long-term service and also reducing energy utilization. In this paper, a new magnetostrictive transducer is proposed and its excitation coil, internal and external magnetic circuit are optimized from the perspective of reducing heat loss. With the help of theoretical and finite element analysis, the response law between key parameters and heat loss of key components are summarized, which provides a basis for reducing heat loss. Finally, according to the optimization scheme, the prototype is processed, and the temperature rise and dynamic output performance of the transducer are tested by constructing an experimental setup. The results show that the transducer has a low temperature rise and good frequency response characteristics, which can provide support for long-time precise actuation on-orbit.

## 1. Introduction

As a kind of intelligent transducer with fast response capability which can achieve more than 1500 ppm magnetostriction [1], giant magnetostrictive transducer (GMT) has been widely used in the manufacture of ultra-precision hydraulic servo valve [2], precision tool positioning [3] and ultrasonic transducer [4] and vibration control [5], etc. Actually, due to the characteristics of high precision and fast response speed, the GMT still attracts researchers from aerospace field attentions [6,7,8]. After all, compared with the traditional motors or actuators, the mechanical structure of the GMT is simple and beneficial for integrated design [9]. However, there are still some questions and difficulties in the applications on-orbit, especially the heat loss which not only reduces energy utilization but also brings burdens to the design of heat dissipation instrument. Furthermore, from another perspective, the heat loss in fact occurs in each process during the energy conversion and transfer from electric energy to mechanical energy. Thus, reducing heat loss has a close relationship with the design of components.

Actually, some remarkable achievements have been obtained by taking a panoramic view of the GMT. As the most important heating part of the transducer, the excitation coil is optimized by Liu to reduce the heating and obtain a better coil shape [10]. However, the coil with the high power shape factor has a limited effect on reducing heat loss. Kwak arranges an air cooling structure inside the coil to reduce the temperature rapidly [11]. Although the effect is obvious, the overall structural size is vastly increased. Moreover, the magnetic energy loss characteristic is discussed by Huang and the measured data indicate that the magnetic energy losses increase rapidly at high frequency and high peak magnetic flux density [12]. In addition, while the magnetic field generated by the coil propagates outward, the ohmic heat generated by the current density could affect the output accuracy of the transducer. Niu considers the eddy current loss induced from both GMM rod and the magnetic yoke and proves that the eddy current loss induced from the yokes makes the output displacement decrease dramatically [13]. Xavier proposes that utilizing specific permanent magnets can achieve magnetic deviation and reduce the thermal effect in the end [14]. A thermal error suppression method using an oil cooling system to control the temperature is proposed by Ju and the research could provide a basis for the application of cylindrical GMT in the precise adjustment of double-nut ball screw preload [15]. Similarly, other researchers put forward their own views on designing a GMT with different aims, such as low eddy current loss characteristics, temperature compensation design, the effect of core geometry on the performance, etc. [16,17,18,19]. However, most of the existing research mainly focuses on the external heat dissipation design and closed magnetic circuit design. Even though the external heat dissipation design could effectively improve the heat dissipation speed and thus extend the service life of the GMT, the auxiliary equipment introduced makes it unsuitable for aerospace applications. Accordingly, even the closed magnetic circuit design could effectively shield the magnetic flux leakage, it does not mean a complete cylindrical yoke is necessary for realizing the shielding. After all, the less the mass of the yoke, the better for aerospace applications.

Thus, in this work, we put emphasis on developing a new GMT which characterizes a self-radiating structure through conducting the low heat loss design, so as to obtain a good working environment. Accordingly, the excitation coil, as an important heat producer, is discussed and re-designed by means of theoretical analysis and numerical simulation for reducing heat loss. Meanwhile, aiming at the design of a closed magnetic yoke, we not only consider the heat loss, but also take the mass of the yokes into account. In this way, it could provide some basis for on-orbit applications in future.

Based on the comments above, the rest of this paper is organized as follows. Section 2 introduces the scheme of the giant magnetostrictive transducer in detail. The physical model is constructed and some key performances are discussed through numerical simulation and FEA simulation for realizing the low heat loss design in Section 3. Finally, a prototype is manufactured, and some experimental tests are carried out to verify the related optimization design in Section 4. Some conclusions are drawn in Section 5.

## 2. Scheme of Giant Magnetostrictive Transducer

**Figure 1:**
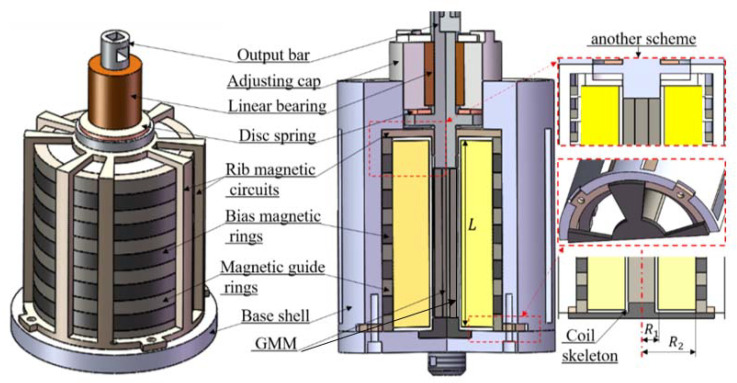
Schematic of the GMT.

The proposed scheme of the new giant magnetostrictive transducer (GMT) is shown in Figure 1. It can be found from the scheme that the giant magnetostrictive material (GMM) rod is located in the center of the coil and is slightly shorter than the axial size of the excitation coil. Different from the traditional scheme in which the biased permanent magnet cylinder is placed between the output rod and the GMM rod, the proposed scheme in which the permanent magnet ring is placed outside the excitation coil is adopted to improve the dynamic output characteristics. It should be noted that the GMM rod is sliced along its length direction for the aim of reducing eddy current loss. The upper part of the output rod adopts the traditional structure [20]. The output rod is designed in contact with the magnetic circuit so that the magnetic line of the driving magnetic field flows through the output rod to the multi-rib magnetic circuit (i.e., external magnetic circuit) as shown in Figure 1, and finally flows back to the GMM rod through the magnetic circuit nested with the base shell (i.e., internal magnetic circuit). The magnetic energy source of the whole transducer includes the constant magnetic field generated by the bias magnetic ring and the driving magnetic field excited by the excitation coil. The excitation coil adopts a double parallel connection, which is beneficial to reduce the heating resistance. Therefore, some optimization problems of the design are as follows: (1) how to reduce the resistance loss of the wiring harness and improve the performance of the coupling inductance; (2) how to reduce the magnetic flux leakage and the distribution of eddy current.

## 3. Physical Model and Finite Element Analysis

### 3.1. Analysis of the Parallel Excitation Coil

**Figure 2:**
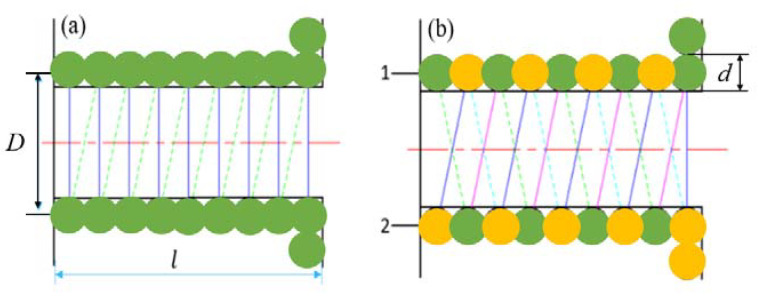
Winding principle of parallel coil: (**a**) coil of single wire, (**b**) parallel interleaved closely wound coil.

As the energy source of the magnetostrictive transducer, the scheme of the coil should not only refer to the requirements of the intensity and uniformity of the drive magnetic field, but also prefer to reduce the unnecessary energy consumption and input lag. Most research focuses on the mechanical efficiency, but those conclusions are not good in reducing the high resistance and high inductance loss of the dense winding coil. While we know the impedance of the excitation coil is directly related to the heat loss, and the heat generated by the coil affects the working time and output precision of the magnetostrictive transducer. Thus, in order to optimize the high resistance of traditional single-enameled wire, a parallel wire scheme is proposed. The winding principle of the two types of coils is illustrated in Figure 2.

**Figure 3:**
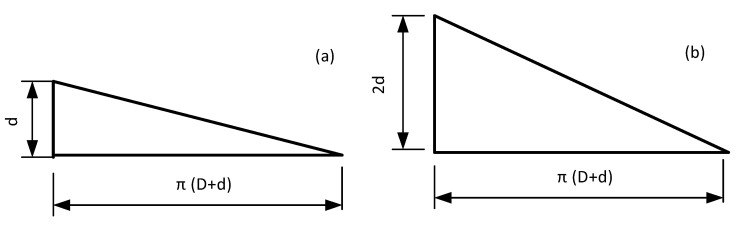
Different cylinder expansion length of wire harness: (**a**) single wire coil deployment structure, (**b**) parallel coil deployment structure.

By expanding any single wire of the coils in the first layer depicted in Figure 2a,b, the single line expansion models depicted in Figure 3a,b can be obtained respectively.

**Figure 4:**
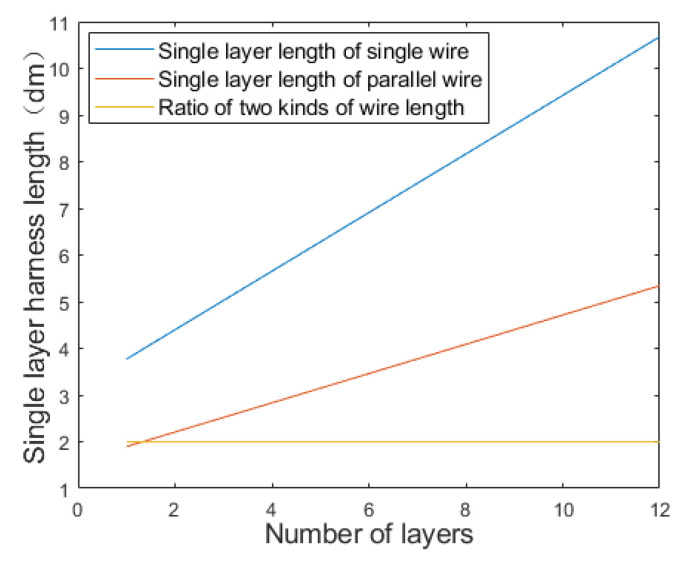
Cylinder expansion length of different coil forms.

**Figure 5:**
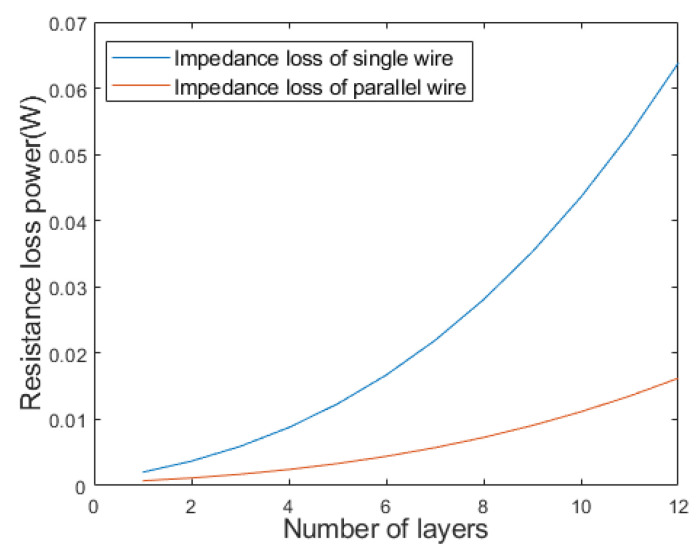
Power loss of different coil forms.

Thus, the unfolding length of single strand of each coil can be calculated by the following relations as,

(1) $$l_{1k}=\frac{1}{2}\sqrt{\pi^{2}{\left(D+\left(2k-1\right)d\right)}^{2}+d^{2}}+\frac{\pi }{2}\left[D+\left(2k-1\right)d\right]$$

(2) $$l_{2k}=\frac{1}{2}\sqrt{\pi^{2}{\left(D+\left(2k-1\right)d\right)}^{2}+4d^{2}}$$

where k is the number of layers of the coil; $l_{1k}$ and $l_{2k}$ are the expansion length of the single-layer wire of the two types of coils, respectively; *D* is the diameter of the coil framework; *d* is the diameter of the wire section. In addition, the length of the wire *l* and the resistance *R* meet the relationship R=ρl/S, where ρ and *S* are the resistivity and the cross-sectional area of the wire, respectively. The resistance loss power P meets the relationship $P\propto R^{2}$, so the relationship between l and P fits $P\propto L^{2}$. Then the wire length and power loss trend with the number of layers can be evaluated through simulation calculation in MATLAB, and the results are shown in Figure 4 and Figure 5.

**Figure 6:**
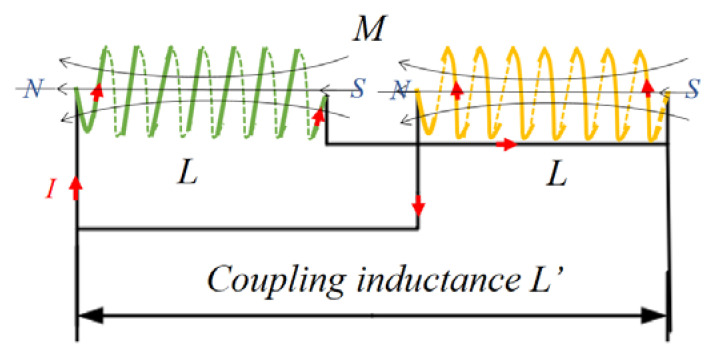
Inductance coupling model of parallel winding.

Figure 4 shows that with the increase of the number of layers, the growth rate of the length of single-layer wires is faster than that of parallel twisted coils with staggered layers. The reason why the ratios look constant is that these two ratios which can be obtained through differentiating the Equations (1) and (2) approximate constants when considering that the diameter of the wire *d* is much smaller than the diameter of the coil framework *D*. Figure 5 shows that with the increase of the number of layers, the cumulative resistance loss power of single wire increases faster. When the layer number is 12, the loss is nearly 4 times of that of the parallel stranded wiring harness. In addition, the low inductance of the wound coil is also important for the magnetostrictive transducer, which in fact has a close relationship with the hysteresis characteristics. The geometric feature of parallel wiring harness is that each layer of coil in the skeleton is a layer of nested helix, thus it meets the inductive coupling model, as shown in Figure 6.

Since the magnetic field direction of the two parallel coils is similar, assume the inductance of the single coil is L, and the inductance after coupling is $L^{\prime }$, then it should satisfy the following equation [21],

(3) $$L^{\prime }=\frac{L^{2}-{\left(M\right)}^{2}}{2L+2M}$$

(4) M=KL

where *K* is the coupling coefficient of mutual inductance coil and *M* is mutual inductance coil. By introducing Equation (4) into Equation (3), we get the following result,

(5) $$L^{\prime }=\frac{\left(1-K\right)L}{2}$$

*K* is the coincidence of the magnetic fields of the two coupled spiral wire bundles, it can be seen from Figure 2b that the coincidence degree of the central magnetic field formed by wire 2 and wire 1 is very high. So, K→1 and $L^{\prime }\to 0$ hold. Then it’s known that the inductance of the proposed exciting coil can be reduced to a very lower level by using parallel nested spiral harness design.

### 3.2. Analysis and Design of Internal Magnetic Circuit

**Figure 7:**
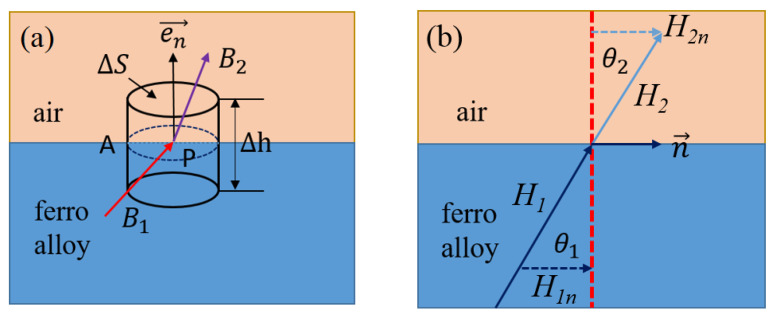
The model of magnetic field line propagation: (**a**) Electromagnetic boundary between two media, (**b**) Refraction relation.

Considering the vector of magnetic field line excited by the coil is alternating in the yoke, the magnetic circuit could produce eddy current and then cause heat loss. In the magnetostrictive transducer structure, the closed-loop magnetic field generated by the ring current always propagates alternately in the air gap and the yoke. Thus, it generally satisfies the boundary conditions of electromagnetic field propagation. As shown in Figure 7, taking any point P at the interface of two propagating media as a flat cylindrical surface surrounding the point, it can reflect the propagation process of magnetic field lines in the closed-loop magnetic circuit.

Let Δh→0, then we can obtain from Gauss Theorem [22],

(6) $$\vec{e_{n}}\cdot \left(\vec{B_{1}}-\vec{B_{2}}\right)\Delta S=0$$

That is $\vec{B_{1e_{n}}}=\vec{B_{2e_{n}}}$. Therefore, the magnetic induction does not decay in the normal direction. In addition, because one of the media is air, no current exists on the interface surface. That is, $J_{S}=0$, and the boundary condition can be transformed into,

(7) $$\vec{n}\cdot \left(\vec{H_{2}}-\vec{H_{1}}\right)=0$$

where $\vec{n}$ is the tangential component and *H* is the magnetic field intensity.

The angle between magnetic field ($B_{2}$ and $B_{1}$) direction and normal direction is θ ($\theta_{2}$ and $\theta_{1}$), then,

(8) $$H_{2}\sin\theta_{2}=H_{1}\sin\theta_{1}$$

(9) $$B_{2}\cos\theta_{2}=B_{1}\cos\theta_{1}$$

As we known that, $B_{2}=\mu_{2}H_{2}$, $B_{1}=\mu_{1}H_{1}$, and *μ* is the relative permeability. Then make the ratio of the two formulas above,

(10) $$\frac{\tan\theta_{1}}{\tan\theta_{2}}=\frac{\mu_{1}}{\mu_{2}}$$

Equation (10) shows that when the magnetic line propagates on the interface of two media, it usually changes its direction and forms a certain refraction angle. If the medium 1 is ferromagnetic material and the medium 2 is air, then $\mu_{2}<<\mu_{1}$, $\theta_{2}<<\theta_{1}$. therefore, $B_{2}<<B_{1}$. It can be seen that the tangential component of ferromagnetic material is greatly weakened after it is propagated through the air. In addition, the exciting current of the magnetostrictive transducer does not change with time, that is, the non-time varying electric field. According to Maxwell’s first equation:

(11) $$\nabla \times \frac{1}{\mu }\vec{B}=\vec{J}+\frac{\partial \vec{T}}{\partial t}$$

where ∇ is gradient operator, J is current density and T is potential shift, respectively. The change rate of displacement current does not produce thermal effect, so it can be ignored in the magnetic circuit. Then if the axial direction is set to *Z* direction in 3D solid space

(12) $$J_{Z}=\frac{1}{\mu }\left(\frac{\partial B_{y}}{\partial x}-\frac{\partial B_{x}}{\partial y}\right)$$

Equation (12) shows the current density in one direction, and it is excited by the magnetic field components in the other two directions, near the coil of the magnetostrictive transducer. That is, when the coil is not in contact with the inner metal magnetic circuit, the current density generated by the tangential component of the magnetic line will be weakened. Thus, based on the weakening mechanism of the tangential component of the magnetic field, the tangential magnetic induction intensity can be evaluated through numerical simulation. α is set as the ratio of the two magnetic induction intensities ($B_{x1}$ and $B_{x2}$), we can obtain

(13) $$\alpha =\frac{B_{x1}}{B_{x2}}=\frac{\frac{B_{x1}}{B_{y1}}}{\frac{B_{x2}}{B_{y2}}}\cdot \frac{B_{y1}}{B_{y2}}=\frac{\tan\theta_{1}}{\tan\theta_{2}}\cdot \frac{B_{y1}}{B_{y2}}$$

In addition, there exists *B_y_*_1_ = *B_y_*_2_, and referring to the trigonometric function transformation formula

(14) $$\tan\left(\frac{\theta }{2}\right)=\frac{1-\cos\theta }{\sin\theta }=\frac{\sin\theta }{1+\cos\theta }$$

Then, based on the formula of refraction ratio, we get,

(15) $$\frac{\sin\theta_{1}}{\sin\theta_{2}}=\sqrt{\varepsilon \mu }$$

Substituting Equations (13) and (14) into Equation (15), the relationship between the electromagnetic refraction ratio and the incident medium parameters can be derived as,

(16) $$4\alpha \cos\theta_{1}{\left(1+\cos\theta_{1}\right)}^{2}=\sqrt{\varepsilon \mu }\left\{{\left(1+\cos\theta_{1}\right)}^{4}-\alpha^{4}\sin^{4}\theta_{1}\right\}$$

where $\theta_{1}$ is the incidence angle, ε is the relative dielectric constant and $\sqrt{\varepsilon \mu }$ is the ratio of the dielectric refractive index.

**Figure 8:**
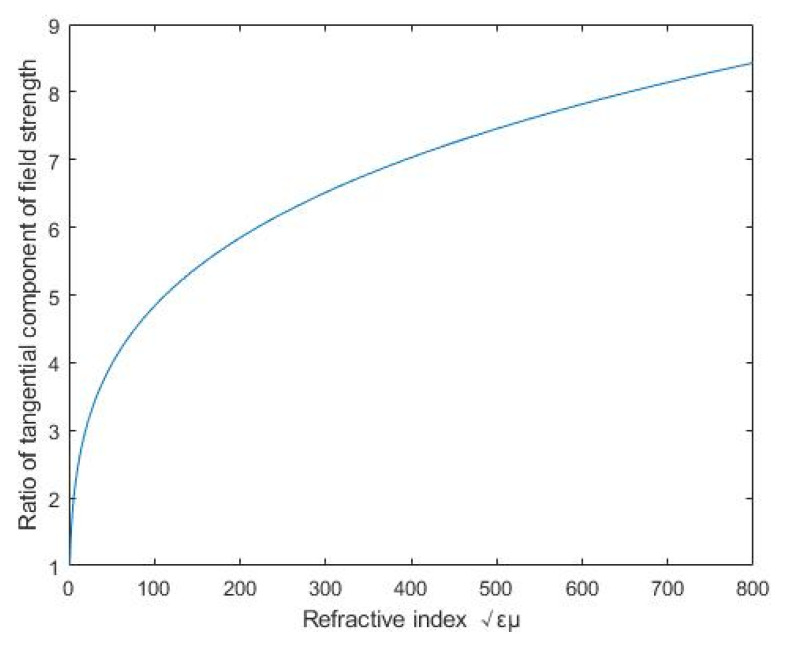
Relationship between field intensity ratio and refractive index.

The incidence angle of the magnetic line in the magnet is defined by all turns of the coil, and it is a small value as a whole, which can be set as 22.5° for simplified calculation. Generally, the relative permittivity of the excellent conductor is about 10, and the relative permeability of ferroalloy is about 240~60,000. There is a great difference in the value of the magnetic line of force entering the magnetic circuit through air or not. When the magnetic circuit is attached to the upper surface of the coil, it can be seen as $\sqrt{\varepsilon \mu }=1$; If not, $\sqrt{\varepsilon \mu }$ is the highest value. Then the Equation (13) is numerically simulated in MATLAB, and the numerical relationship between tangential field intensity ratio and refractive index is drawn in Figure 8.

**Figure 9:**
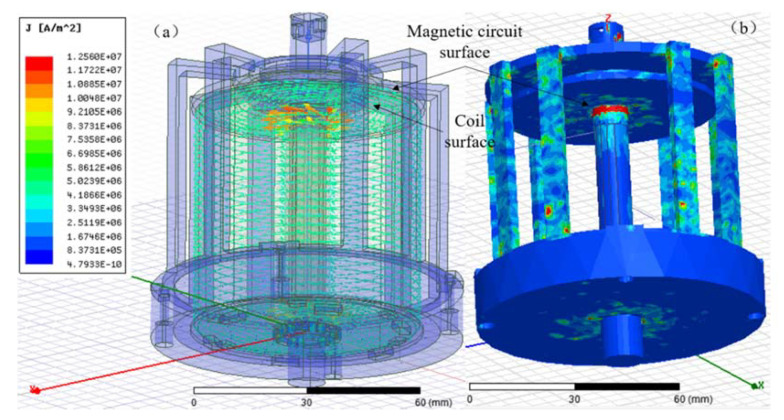
Current density simulation: (**a**) Arrow diagram of current density distribution, (**b**) Hysteresis ohmic loss diagram.

Figure 8 shows the relationship between the field intensity ratio and the refractive index. However, in fact, the trend between the field intensity ratio and the refractive index changes gradually, but abruptly. That is because the effective value points on the curve of Figure 8 should be (1, 1) and (800, 8.429), which reflect whether the magnetic circuit is close to the excitation harness or not. The number 8.429 reflects the inherent effect of air gap on reducing current density. In order to further verify the accuracy of numerical trend, the current density distribution is obtained by ANSYS Maxwell and the results are depicted in Figure 9.

**Figure 10:**
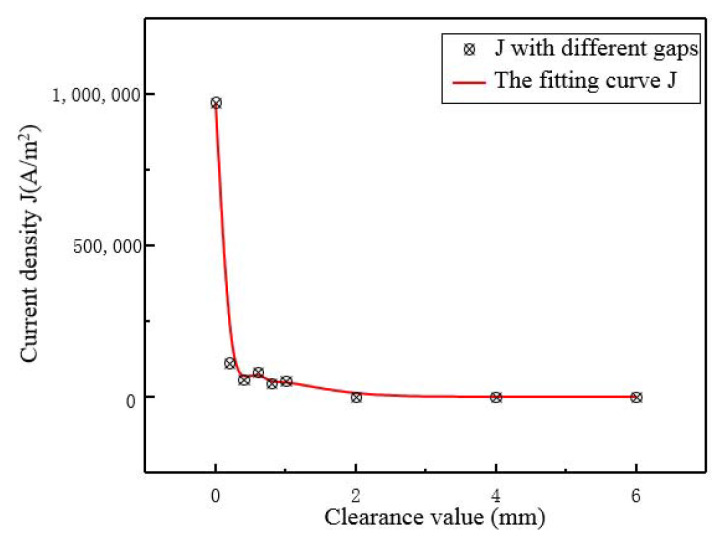
Maxwell current density simulation results with different spacing.

The eddy current loss in Maxwell simulation firstly verifies the skin effect at the inflection point and small cross-section, and then we adjust the distance between the coil and the upper surface of the magnetic circuit to obtain the current density simulation results, as shown in Figure 10.

It can be seen that the peak current density in the direction of the central axis always occurs in the relatively stable region around the center of the output rod. When there is no gap, the peak value of current density is the largest value, but then the change trend of current density is small with the gap increasing from 0.2 mm to 1 mm. The ratio of the maximum current density of the laminating structure to the peak current density of the structure with gap is about 9, and the relative error is 6%, compared with the progressive refractive index 8.429. Therefore, the conclusion can be drawn: in order to reduce the heat loss caused by the induced current of the magnetic circuit, a suitable isolation gap is necessary which means that retaining a certain distance between the magnetic circuit and the excitation coil should be taken into consideration during the design of transducer from the perspective of the electric field and current density generated by the changing magnetic field. Thus, for the convenience of manufacture, the value can be taken as 1 mm.

### 3.3. Analysis and Design of External Magnetic Circuit

**Figure 11:**
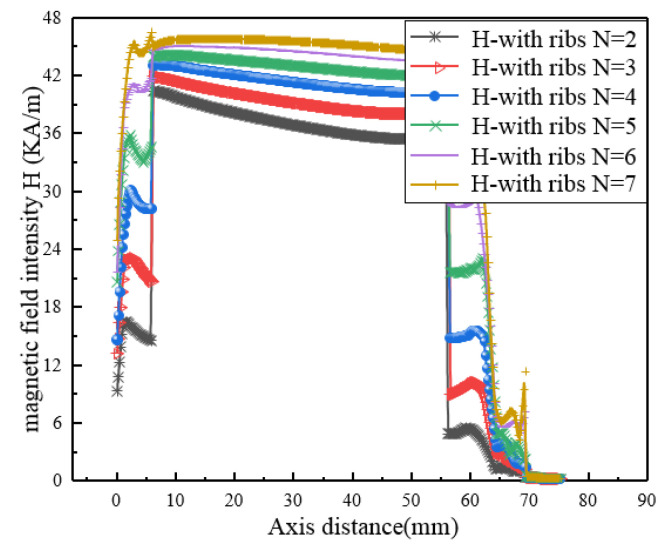
*H* of different magnetic circuits.

**Figure 12:**
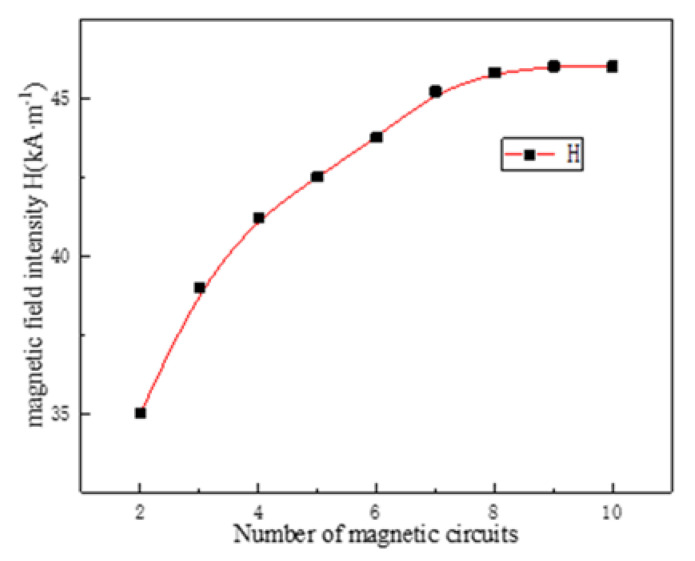
Field strength growth trend.

**Figure 13:**
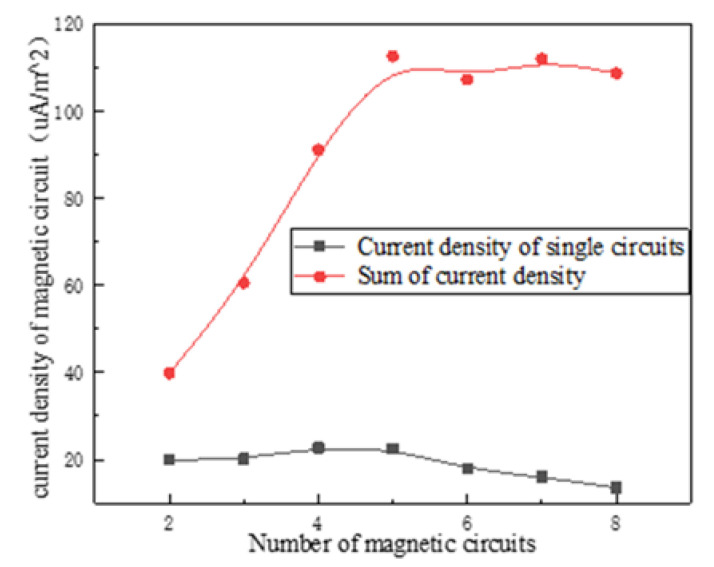
*J* of different circuits.

**Figure 14:**
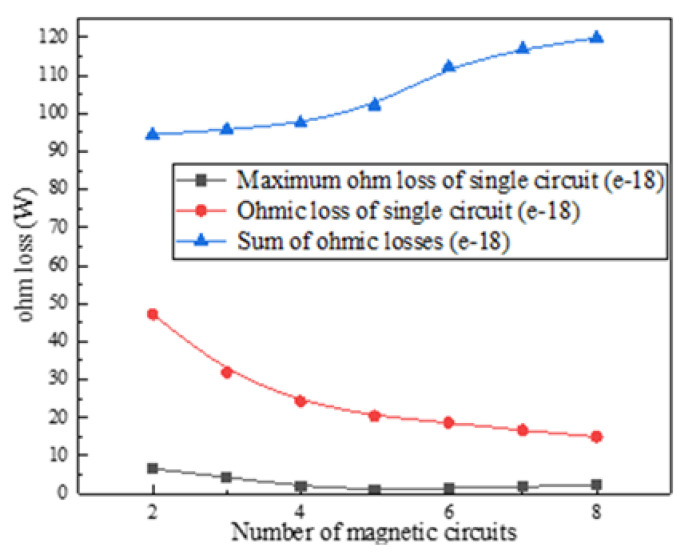
Ohmic loss of different circuits.

After the internal magnetic circuit of the magnetostrictive transducer is defined, the current density in the output rod can be reduced, which is conducive to reducing the influence of temperature rise on the performance of magnetostrictive materials. However, the overall temperature distribution of the transducer always presents a trend of high inside and low outside due to the existence of the excitation coil, so a lower temperature outside the transducer is conducive to improving the temperature gradient so as to form the condition for rapid heat dissipation. Even a fully closed-loop magnetic circuit is beneficial to reduce magnetic flux leakage, it also increases the induction loss and has no advantages in increasing the heat dissipation area and improving the temperature gradient. Therefore, the design of the embedded multi-rib magnetic circuit is adopted for realizing these functions above. In order to study the relationship between the multi-rib magnetic circuit and the performance of the transducer, numerical simulation is carried out to study the relationship between the magnetic field intensity, the current density of the magnetic circuit, the ohmic loss and the number of magnetic circuits under the same excitation current. The results are shown in Figure 11, Figure 12, Figure 13 and Figure 14.

It can be found from Figure 11 that the magnetic field intensity *H* and uniformity of the driving magnetic field in the central axis of the magnetostrictive transducer generated by the excitation coil are the best, but the non-uniformity is also presented at the joint points between the components. In addition, with the increase of the number of external magnetic yoke ribs, the magnitude and uniformity of the magnetic field strength are gradually increased, but the increased amplitude is gradually weakened especially when 7 magnetic yoke ribs are adopted (N7). Meanwhile, Figure 12 shows more clearly that the relationship between the magnetic field strength and the magnetic circuits. When the number of magnetic circuits goes from 8 to 10, the field strength along the axis is basically in the maximum state, which means the magnetic field gradually becomes saturated.

Figure 13 and Figure 14 show that the current density of a single magnetic yoke rib increases first and then decreases, but the total current density of different magnetic circuits tends to be saturated finally; In addition, with the increase of the number of magnetic circuits (i.e., the magnetic yoke ribs), the ohmic loss in a single circuit decreases gradually, but the total ohmic loss increases continuously. Finally, eight magnetic yoke ribs are selected in the scheme in consideration of the requirements of driving magnetic field strength and reducing the induction loss.

## 4. Experimental Study of the Transducer

### 4.1. Experimental Setup of the Transducer

**Figure 15:**
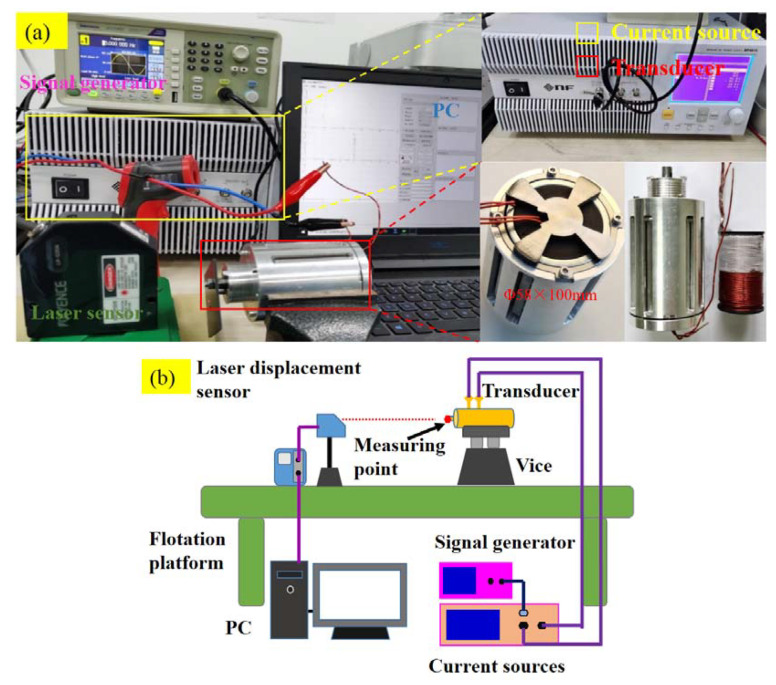
Experimental setup of the transducer: (**a**) experimental setup, (**b**) block diagram of the setup.

The experimental setup for testing the magnetostrictive transducer that is performed for the actuator mode mainly consists of a signal generator (Tektronix, AFG1062, Tektronix, Inc. Beaverton, United States), a linear bipolar current source (NF, BP4610, NF Co. Yokohama, Japan), a laser displacement sensor (Keyence, LK-G30, Keyence Co., Osaka, Japan), an infrared thermometer (UNI-T, UT302, UNI-T Co., Dongguan, China) and the proposed giant magnetostrictive transducer. The signal generator firstly generates the expected excitation signal. Then the signal is amplified by the bipolar current source which can display the actual current value in the meanwhile, and subsequently is applied to the transducer. Finally, the laser displacement sensor is utilized to collect the displacement response of the prototype and the infrared thermometer is used to collect temperature information. The experimental setup is shown in Figure 15.

### 4.2. Temperature Characteristic Tests

**Figure 16:**
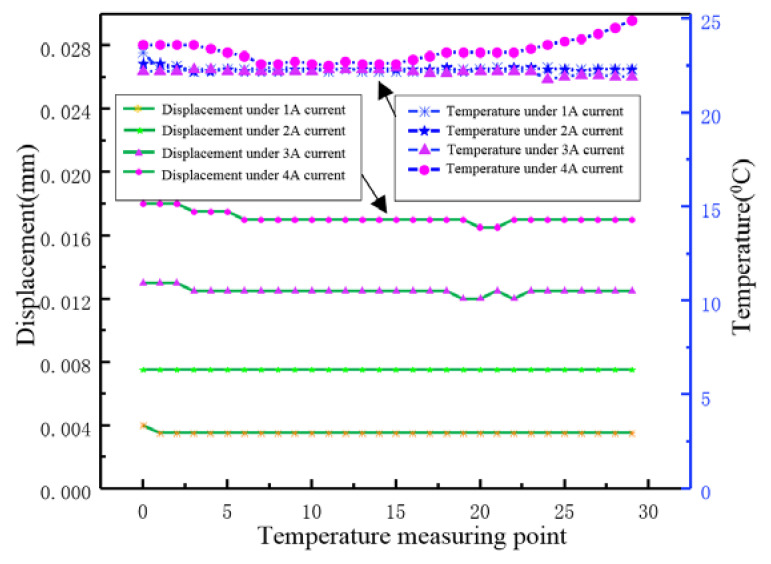
Static thermal expansion test of 1A to 4A DC.

In view of the low heat loss design, the static thermal expansion tests are carried out and the results are shown in Figure 16. These results are obtained through equal interval measurement. In other words, we collect the temperature and the response stroke every 10 s, under the different DC from 1A to 4A. Thus, it can be seen that the proposed transducer does not present obvious thermal expansion during more than five minutes of temperature rise tests. Under the 1A driving current, the output and temperature response of the transducer are both maintained at a lower level, which means the heat dissipation is less and relatively stable. As the increasing of driving current, the output response becomes larger, but the overall temperature response is basically constant. When the current is 4A, a certain temperature fluctuation presents, but no obvious thermal expansion generates according to the displacement output curve. Thus, it can be said that the prototype with low heat loss design has achieved good effects.

**Figure 17:**
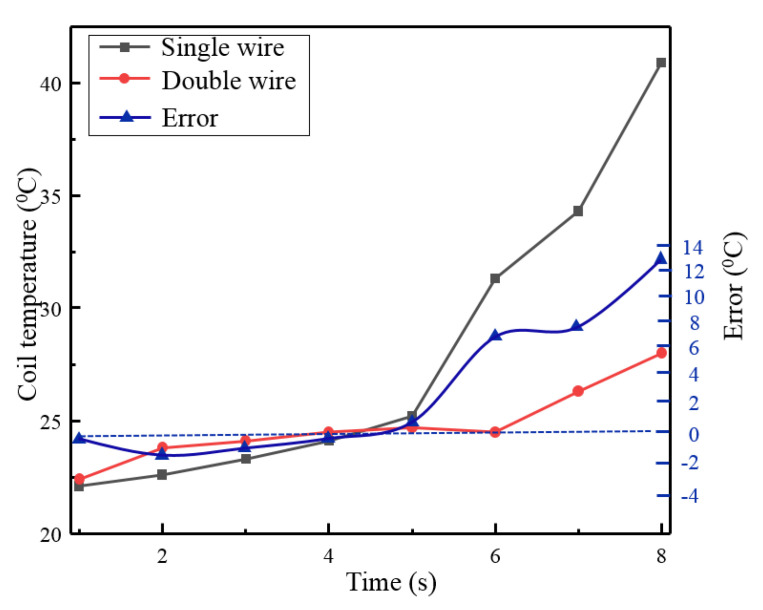
Comparison of temperature rise between single coil and parallel coil.

In addition, in order to further verify the effectiveness of the proposed excitation coil, we compare the heat loss characteristics of the two-wire coil with a single wire coil. For simplicity, 6A inputting current is selected for the coil heating tests and the temperature rise curve are depicted in Figure 17. It shows clearly that these two types of coils present temperature rise after 5 min, but the rise speed of the single wire coil is much faster than that of the double wire. The temperature of our proposed coil is only 27.2 °C after 8 min of power on, while the temperature of the single wire coil exceeds 40 °C. Thus, it can be said that the heat loss of the double wire coil is lower than that of the single wire, which, in fact, is consistent with the theoretical trend of coil power consumption.

### 4.3. Frequency Response Tests

**Figure 18:**
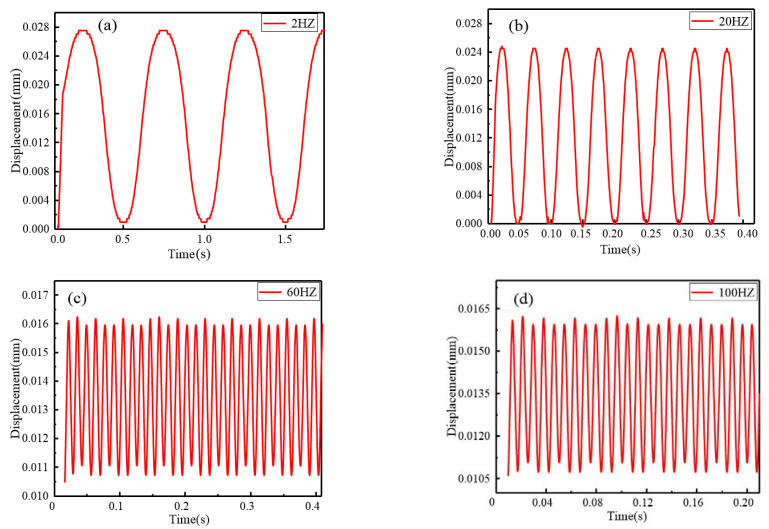
Displacement response: (**a**) 2 Hz, (**b**) 20 Hz, (**c**) 60 Hz, (**d**)100 Hz.

**Figure 19:**
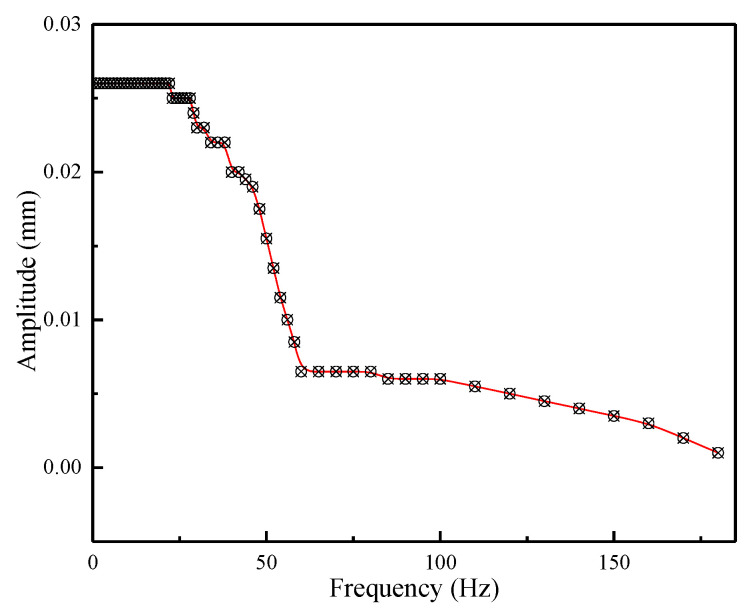
Amplitude frequency characteristics of the transducer.

A wide working bandwidth of the transducer can make itself well adapted to work in different conditions. Figure 18 shows the displacement output responses at different frequencies when giving the prototype a sinusoidal current with same amplitude of 8 A and different frequencies. It is obvious that the proposed transducer is able to generate the corresponding responses according to the applied driving signals. Meanwhile, it also reflects the transducer owns a stable output capability under 20 Hz driving frequency, which is in fact enough for aerospace applications. Furthermore, the amplitude-frequency curve is depicted in Figure 19 to proclaim the dynamic response in more detail. We can find that nearly no influence can be taken to the output displacement by the driving frequency below 25 Hz. In other words, the transducer can work well until 25 Hz which means that the transducer is able to realize nearly 26 μm displacement output. Then the output decreases quickly until 60 Hz and the displacement response attenuates from 26 μm to 6 μm. Finally, a gentle downward trend is presented as the driving frequency increases. Overall, it conforms to the attenuation law of the giant magnetostrictive transducer.

### 4.4. Resolution Tests

**Figure 20:**
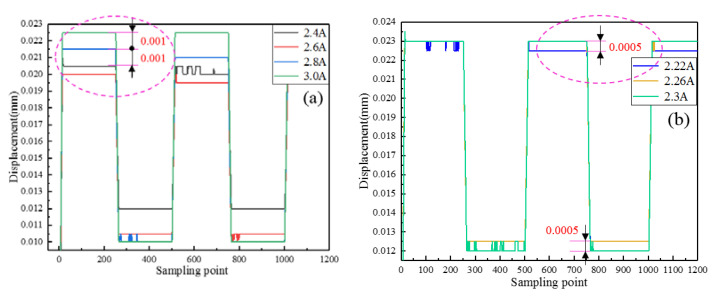
Resolution tests results: (**a**) 0.2A impulse response, (**b**) 0.04A impulse response.

Magnetostrictive material has inherent hysteresis nonlinearity characteristics. Therefore, the resolution of the transducer on one hand reflects its output precision, on other hand reflects the effect of low heat loss design on reducing the nonlinear output performance. The test results are drawn in Figure 20. Figure 20a shows the results driven by square wave current with 2A bias current and 0.2A interval, while Figure 20b illustrates the results driven by square wave current with 2A bias current and 0.04A interval. Then it is obvious that similar square wave responses are presented under the different driving currents. Accordingly, the observable displacement variation is 1 μm and 0.5 μm, respectively. In other words, the transducer possesses the corresponding dynamic output capability. Thus, it can be said that the test resolutions can be up to 1 and 0.5 μm, respectively. Actually, the resolution can be further improved by adopting a higher performance displacement sensor. However, overall, it provides the possibility for precision applications on-orbit.

### 4.5. Hysteresis Characteristics

**Figure 21:**
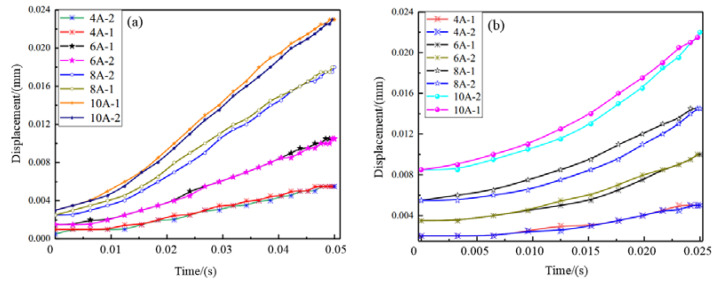
Hysteresis loop tests: (**a**) 10 Hz sinusoidal excitation, (**b**) 20 Hz sinusoidal excitation.

The hysteresis characteristics of the giant magnetostrictive transducer is important and can be affected by the driving frequency. Thus, the hysteresis characteristics in the stable response bandwidth below 20 Hz are studied, and the results are shown in Figure 21. It should be noted that the “-1” and “-2” in the figure legend mean the displacement responses under the rising section and descending section of the sinusoidal excitation current. It can be found that the relatively small hysteresis loop areas are presented. That means less useless magnetic energy in a magnetization cycle is consumed. Furthermore, the hysteresis loop area becomes large when the driving frequency increases. Especially when the excitation current with a frequency of 20 Hz reaches 8 A, the expansion area of hysteresis loop increases obviously. In addition, from the overall trend, the higher the driving frequency, the larger the hysteresis loop. Accordingly, it could improve the difficulty of closed-loop control during precision actuation.

## 5. Conclusions

A new design of magnetostrictive transducer with low heat loss is proposed for space applications in this article. Aiming at the problem of high coil resistance and inductance, a parallel coil is designed and the advantages are verified by the numerical simulations. In addition, the internal and external magnetic yokes and heat transfer structure are constructed and analyzed by theory and FEA simulation. And based on these conclusions, the details of the GMT are defined. Finally, a prototype is processed and the experimental tests are carried out. The results prove that the temperature rise speed is slower due to the adoption of a low heat loss design. In addition, the stable working bandwidth is up to 25 Hz and the maximum amplitude is about 26 μm. Meanwhile, the resolution tests and the hysteresis loss tests are also conducted, and the results show excellent output performance in low frequency, which is especially suitable for aerospace applications. In addition, due to the limited test conditions, further experimental studies can be carried out in future.

## Data Availability

The data presented in this study are available on request from the corresponding author.
